# Global maps of transcription factor properties reveal threshold-based formation of DNA-bound and mobile clusters

**DOI:** 10.1126/sciadv.ady3909

**Published:** 2026-02-27

**Authors:** Sadia Siddika Dima, Meghan A. Turner, Hernan G. Garcia, Gregory T. Reeves

**Affiliations:** ^1^ Department of Chemical Engineering, Texas A&M University, College Station, TX 77843, USA.; ^2^ Biophysics Graduate Group, University of California, Berkeley, Berkeley, CA 94720, USA.; ^3^ Department of Molecular and Cell Biology, University of California, Berkeley, Berkeley, CA 94720, USA.; ^4^ Department of Physics, University of California, Berkeley, Berkeley, CA 94720, USA.; ^5^ Institute for Quantitative Biosciences-QB3, University of California, Berkeley, Berkeley, CA 94720, USA.; ^6^ Chan Zuckerberg Biohub – San Francisco, San Francisco, CA 94158, USA.; ^7^ Faculty in Genetics and Genomics, Texas A&M University, College Station, TX 77843, USA.

## Abstract

The relationship between bulk transcription factor concentration and DNA binding has been a central question in gene regulation for decades. Recent studies propose that DNA-bound transcription factor hubs, or clusters, aid in fast and precise transcriptional interpretation. Using live imaging techniques, we quantify the concentration, binding, and mobility of the morphogen Dorsal (Dl), both in bulk and in clusters, in *Drosophila* blastoderm embryo. Our experiments encompass multiple length and timescales, allowing us to obtain a nucleus-wide view of the mechanism connecting hub formation to bulk Dl concentration. Our results show that previously unobserved, slowly moving clusters of Dl are present, in addition to the expected populations of freely mobile and DNA-bound Dl. Furthermore, both mobile clusters and DNA-bound Dl appear only once a threshold concentration in the nucleus is surpassed, a behavior consistent with liquid-liquid phase separation. Thus, our work elucidates how bulk transcription factor concentration dictates the formation and spatiotemporal changes of different populations needed for gene regulation.

## Teaser

DNA-bound and mobile clusters of transcription factor are formed above a threshold in the bulk concentration.

## INTRODUCTION

Cell fate determination and maintenance in metazoans depend on the accurate spatiotemporal regulation of gene expression. The binding of transcription factors (TFs) to regulatory motifs in the DNA in eukaryotes, and thereby the target gene expression, is dependent on a plethora of factors including its concentration, binding affinity, chromatin accessibility, and protein-protein interactions (*1*–*4*). Therefore, bulk concentration, a parameter commonly estimated from the fluorescence intensity of fixed or live imaging, cannot serve as a direct proxy to TF activity; instead, measurements of TF-DNA binding, in relationship to the bulk concentration, are required. Recent studies also suggest that TFs form DNA-bound clusters (or hubs) of elevated concentration [reviewed in (*5*, *6*)], potentially mediated by intrinsically disordered regions (IDRs) (*7*–*9*). It has been proposed that these clusters represent liquid-liquid phase-separated droplets, although that suggestion remains a matter of debate (*5*, *6*). In *Drosophila*, the formation of TF hubs has been found to facilitate binding to low-affinity sites by increasing the local concentration of TFs (*10*–*12*). These hubs aid in transcriptional burst induction (*9*) and function in a gene-specific or enhancer-specific manner (*13*). However, the mechanisms connecting bulk TF concentration, on the micrometer scale, to DNA binding and hub formation, on the nanometer scale, remain poorly understood.

Dorsal (Dl), a *Drosophila* homolog of mammalian nuclear factor κB, acts as a morphogen to pattern the dorsal-ventral (DV) axis of the *Drosophila* blastoderm–stage (1- to 3-hour old) embryo, regulating hundreds of genes (*14*–*16*). Dl is one of the earliest known morphogens (*17*) and one of the best characterized sequence-specific TFs (*18*). Previous work using fluorescent imaging of live and fixed embryos indicated that the Dl gradient has a Gaussian-like shape with the highest level of Dl present on the ventral side of the embryo (*14*, *19*). The level of Dl oscillates in a progressively increasing saw-tooth pattern during blastoderm nuclear cycles (ncs) 10 to 14 (*14*, *20*). This temporal variation has been proposed to ensure the accurate dynamic expression patterns of its target genes (*14*). However, modeling efforts that take spatiotemporal variation of the Dl gradient as the only input fail to predict expression pattern and dynamics of all the target gene domains [reviewed in (*21*, *22*)]. Hence, knowing the bulk nuclear intensity of Dl is not sufficient to explain patterning. Intriguingly, recent groundbreaking work has shown that the activity of many TFs, including Dl, is also influenced by the presence of clusters (also referred to as hubs, aggregates, droplets, or condensates), and the enrichment of Dl clusters has been observed at the site of transcription in both fixed and live embryos (*13*, *23*). However, how the dynamic Dl gradient dictates the formation of these populations of clusters remains unexplored.

Thus, to understand the transcriptional interpretation of the Dl gradient, knowing the dynamics of Dl bulk nuclear concentration is an important, necessary piece, but it is not sufficient by itself. In the same way, knowing the cluster formation dynamics at specific enhancers is also required for a detailed view of gene regulation (*13*), but it is again not the full picture. Measurements of different populations of Dl—such as the freely diffusing population, the DNA-bound population, clusters of Dl—and, in particular, how they relate to each other in dose/response maps, must be considered. Therefore, we used advanced live imaging and analysis techniques, such as raster image correlation spectroscopy (RICS) (*24*–*29*), a type of scanning fluorescence correlation spectroscopy (*30*–*32*), and single particle tracking (SPT) of super-resolution images of an endogenously tagged Dl. These experiments, which span a wide range of length and timescales, indicate the presence of three different populations of Dl: freely moving Dl, DNA-bound Dl, and a previously unobserved population of slowly moving clusters of Dl, which are distinct from the DNA-bound transcription hubs of Dl that have been previously reported (*13*, *23*). A recent study similarly observed the formation of mobile clusters of two other blastoderm patterning factors, Bicoid (Bcd) and Capicua (Cic), and proposed these mobile clusters to be an efficient search strategy for regulatory regions (*33*). Thus, mobile cluster formation might be a common mechanism for the accurate transcriptional interpretation of morphogen gradients. Our results further suggest that the total nuclear concentration of Dl must reach a threshold of roughly 30 nM before DNA binding occurs and before the slowly moving clusters of Dl are formed, potentially indicating an ultrasensitive process or, alternatively, a liquid-liquid phase separation (LLPS) process. Ultimately, our results suggest that the relationships of the different populations of Dl in the nucleus, including DNA-bound as well as slowly moving clusters, must be taken into account to understand transcriptional regulation on a tissue-wide level. We suggest that the importance of these relationships is also extended to other TF systems.

## RESULTS

### The Dl nuclear concentration and binding is dynamic throughout the blastoderm stage

**Fig. 1.**
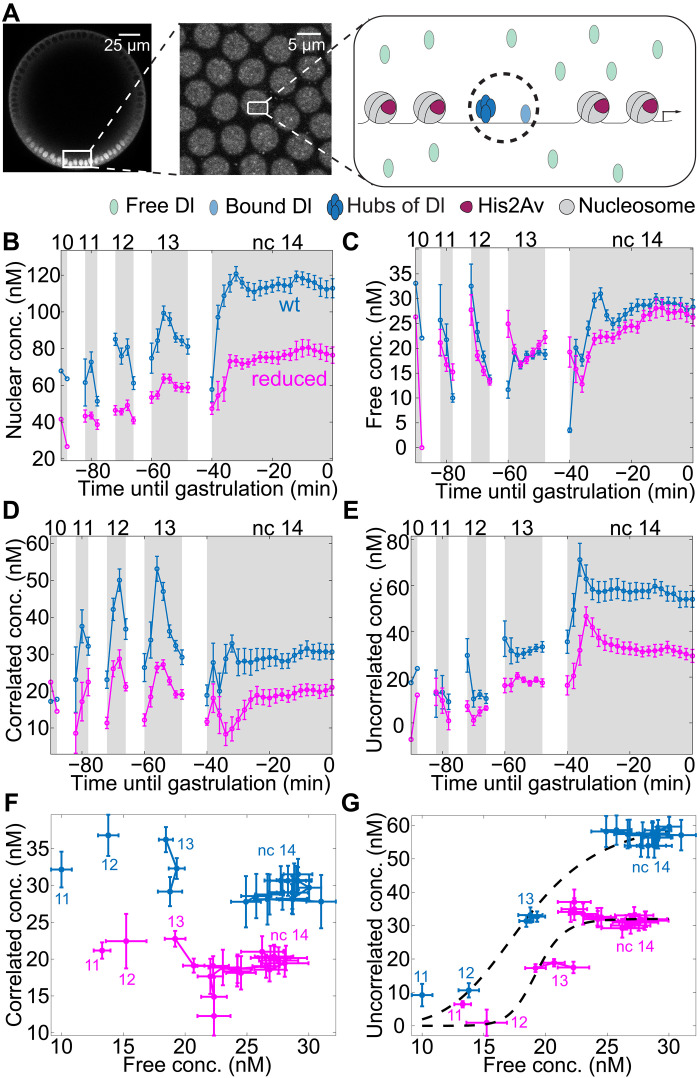
Temporal variation of biophysical parameters of Dl in a fly line having wt level of Dl and in a line where the level is reduced (reduced) in the ventral-most nuclei. (**A**) Representative image showing the Dl gradient (wt) and RICS acquisition in the ventral-most nuclei. We expect to see three populations: free, DNA-bound, and clusters of Dl as depicted in the figure. Populations inside the dashed circle are identified as similar (correlated in this case) using RICS. (**B** to **E**) Dynamics of different pools of Dl from nc 10 until gastrulation including total nuclear concentration (B), free concentration (C), correlated concentration (D), and uncorrelated concentration (E). The dynamics of the free population of Dl from nc 10 until gastrulation show only minor differences between the two fly lines compared to the other pools. Curves: mean values (*n* = 8 for wt embryos, *n* = 8 for reduced embryos). Error bars, SEM. (**F** and **G**) Dose/response map between free and the low-diffusivity populations including correlated population (F) and uncorrelated population (G).

Studying the dose/response behavior between the bulk concentration of a TF and its DNA binding interactions, which can provide important insights into gene regulation, requires measurements at a range of concentrations. To do so, we leveraged the fact that the Dl gradient has dynamically varying levels over ncs 10 to 14. We made use of a CRISPR-edited *dl-mNeonGreen* (*dl-mNG*) construct in which the *mNeonGreen* was inserted scarlessly in-frame just before the stop codon of the endogenous *dl* locus [referred to as the “wild-type” (wt) construct]. At two copies, this scarless CRISPR edit is viable and results in similar target gene expression to wt. We imaged the ventral-most nuclei (Fig. 1A) from nc 10 to gastrulation in embryos collected from mothers having one copy of the Dl-mNeonGreen wt construct and one copy of His2Av–red fluorescent protein (RFP) (to mark the DNA). This line is used in the rest of our experiments unless mentioned otherwise. Our time series image acquisitions (movie S1) were compatible with RICS analysis, which allowed us to quantify the temporal dynamics of the total nuclear concentration and binding of Dl (see Materials and Methods and fig. S1). For each cycle, the imaging was started at early interphase and continued until the onset of prophase indicated by chromatin condensation (*34*). We found that the absolute nuclear concentration of Dl increases from one nc to the next from ~60 nM in nc 10 to ~120 nM in nc 14 (blue wt data in Fig. 1B), consistent with the increase in Dl gradient amplitude over time observed previously (*14*, *19*, *20*, *35*). All concentration values are background subtracted (see Materials and Methods). During each nc, the nuclear concentration of Dl increases in the beginning of interphase as Dl enters the nucleus after mitosis and then drops abruptly as the nuclear envelope breaks down before the next mitosis (*14*, *20*). The long duration of nc 14 compared to the other ncs allows the nuclear concentration to reach a steady-state value of ~120 nM.

According to our RICS measurements, the total nuclear concentration of Dl can be divided into two pools: freely diffusible Dl and slowly moving Dl (Fig. 1A; see Materials and Methods) (*26*, *29*, *36*). The concentration of the freely diffusible pool of Dl was found to be maintained between roughly 20 and 30 nM during the blastoderm stage, reaching a steady state of ~30 nM during mid-nc 14 (Fig. 1C).

The “slowly moving” pool of Dl, which we defined as having a zero or nearly zero diffusivity in our RICS analysis, is likely to be composed of Dl monomers or clusters bound to DNA. Both of these species would have a nearly zero diffusivity and thus are indistinguishable in this method, being lumped together into this pool (indicated by a dashed circle around them in Fig. 1A). This pool could also, in principle, contain slowly moving Dl with diffusivities significantly less than 1 μm^2^/s, as diffusivities that low would be difficult to distinguish from zero. Therefore, to dissect the slowly moving pool further, we performed cross-correlation RICS (ccRICS) by cross-correlating the fluctuations between Dl-mNG and His2Av-RFP (see Materials and Methods) (*26*, *29*, *36*). We found that the population of Dl in the slowly moving pool can be subdivided into the pool of Dl that cross-correlates with His2Av-RFP [likely representing a DNA-bound population (*37*, *38*); referred to as the “correlated” pool henceforth], and the pool that does not (referred to as the “uncorrelated” pool) (*36*). The concentration of Dl in the correlated pool peaks in ncs 12 and 13 then decreases in nc 14 to a steady state of roughly 30 nM (blue data in Fig. 1D; see also fig. S2A). From these measurements alone, it is unclear what constitutes the remainder of the slowly moving pool (not correlating with His2Av). Given the recent discovery of slowly moving clusters of Bcd and Cic (*33*), we hypothesize that the uncorrelated population we observe possibly includes slowly diffusing oligomeric clusters of Dl (the presence of which is tested in the following sections). The concentration of this uncorrelated pool, determined from the difference between the concentrations of immobile Dl and correlated Dl, increases over time (blue data in Fig. 1E; see also fig. S2B).

Both subpopulations of the slowly moving pool are likely due to Dl-binding interactions (either to DNA or to some unidentified structures). Therefore, we asked whether these binding interactions follow typical models of protein binding, such as TF/DNA interactions. These models are often loosely based on binding affinity and are typically saturable functions of the free concentration, such as Hill functions (*36*, *39*–*43*). In reality, the relation is complicated by the influence of several factors, including binding affinity, chromatin accessibility, and protein-protein interactions (*1*–*4*). Our measurements of the different subpopulations of Dl afford us the opportunity to test these models of DNA binding by constructing dose/response relationships between the correlated or uncorrelated concentrations of Dl and the free concentration. To construct these relationships, we included only the data points after the nuclear concentration peaked, which allowed for binding interactions to approach equilibrium. As it appeared that the later/longer ncs took longer to reach peak nuclear concentration (see Fig. 1B), we excluded fewer points in the earlier/shorter ncs. Accordingly, all data points for nc 10 were avoided because of its short duration and the first 2, 3, 4, and 5 time points in ncs 11 to 14, respectively (as indicated in Fig. 1, B to E) were avoided. Unexpectedly, we found that the binding of Dl to DNA, represented by the correlated concentration, is independent of the free concentration (blue data in Fig. 1F) rather than an increasing function of free concentration (as would be expected). This independence of the free Dl concentration suggests that a simple binding affinity model fails to predict DNA binding and, thus, transcriptional response. In contrast, the uncorrelated pool (blue data in Fig. 1G) could possibly be explained with a dose/response relationship between the uncorrelated concentration and the free concentration. To illustrate this, we fit a fourth-order Hill function to the data (see black dashed curve in Fig. 1G; see also Materials and Methods). We noted that, specifically within nc 14, the uncorrelated concentration does not change with variations in the free concentration. This apparent lack of relationship within nc 14 could result from saturation of the uncorrelated pool.

### Reduced levels of Dl result in an altered dose/response relationship

To further test the apparent dose/response relationship, approximated by a Hill function, we asked whether a reduction in Dl levels would result in data points that fall on the same dose/response curve. To address this question, we used a Dl-mNG line that has the same protein sequence but has a 50% reduction in Dl levels (referred to as “reduced”). We had previously shown that, compared to wt, this fly line has half the *dl* mRNA level, resulting in half Dl protein level, and thereby altered target gene expression pattern. These differences are caused by the presence of a DsRed selectable marker in the 3′ untranslated region (*44*). To compare the nuclear levels at different DV positions, we imaged the embryo cross section. We found that the spatial gradient was reduced to half globally in the reduced line (fig. S2C). This suggests that the results in the ventral-most nuclei hold for the nuclei in other DV locations. Using this reduced fly line is advantageous because we are able to reduce the nuclear Dl levels across the whole time course (magenta in Fig. 1B) while holding the DV location and, hence, the Toll-receptor–associated signaling (*45*–*47*), constant. However, since the reduced line has a very low viability (*44*) when there are two copies of tagged Dl, all the results for both the wt and reduced lines are performed on the embryos having one copy of tagged Dl to ensure that the observed biophysical parameters can be compared and are not affected by the reduced hatch rate caused by the genomic edits. As a control, we also imaged wt embryos with two copies of Dl-mNG (*n* = 8), and the total nuclear concentration is doubled compared to embryos with one copy, as expected, while the distribution of the populations remains the same (fig. S3).

As the only difference between the wt embryos and the reduced embryos is the total Dl concentration (and not Dl protein sequence or DV location), the base expectation would be a reduction in the concentrations of all pools of Dl. While the two slowly moving populations follow expectations and are generally decreased in the reduced line (magenta data in Fig. 1, D and E), the free concentration in the reduced Dl line is, unexpectedly, almost identical to that of the wt line (compare magenta and blue curves in Fig. 1C).

If the uncorrelated concentration is truly a simple function of the free concentration, we would expect the data points for both fly lines to fall on the same dose/response curve (uncorrelated concentration versus free concentration). However, we found that the data points of the reduced embryos were shifted with respect to those from the wt embryos (magenta data in Fig. 1G) rather than falling on the same dose/response curve. To illustrate this, we fit another fourth-order Hill function to the data from the reduced line (see dashed curve in Fig. 1G). Hence, a simple dose/response relationship, such as a Hill function dependent only on free Dl concentration, fails to explain the observed behavior, which implies that both subpopulations of slowly moving Dl are not primarily functions of the available (free) Dl concentration.

Given that the free concentration is the same between the two lines, although the total concentration differs, our data suggest that there might exist a critical threshold of total nuclear Dl concentration (~30 nM). Below this threshold, all Dl would be freely diffusing, while any Dl concentration above this threshold becomes allocated to the slowly moving pools (DNA-bound and/or mobile clusters), keeping the free Dl pool unchanged. Such a threshold could be the result of a highly ultrasensitive response to the total Dl concentration (not the free Dl concentration), calling into question whether models of affinity-based binding are appropriate for Dl. Although the dose-response relationship of the wt and reduced lines can separately be explained by the affinity-based models (see dashed lines in Fig. 1G), we need to consider some other model to explain the threshold-based behavior. One explanation of this threshold-based behavior would be an LLPS-driven process.

### Free concentration remains roughly constant, while other populations are dictated by bulk concentration along the Dl gradient

**Fig. 2.**
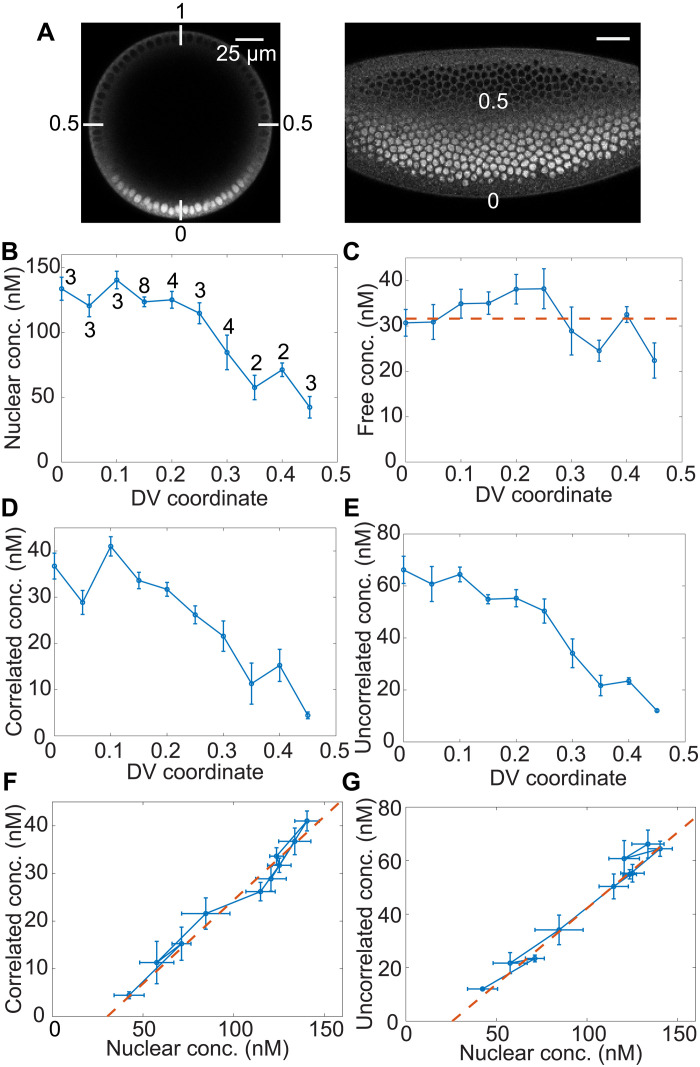
Spatial variation of biophysical parameters of Dl mid nc 14. (**A**) Snap shots of Dl-mNG in nc 14 embryos, illustrating the relationship between Dl nuclear intensity and DV coordinate in the embryo cross section (left) and surface (right). The image in the left panel is the same as that in Fig. 1A (left) for the ease of comparing the position of the region of interest in the DV axis across different experiments. (**B** to **E**) Variation of different pools of Dl in ventral to lateral regions of the embryo including total nuclear concentration (B), free concentration (C), correlated concentration (D), and uncorrelated concentration (E). The concentration of free Dl is only a weak function of DV location. Curves: mean values. Error bars, SEM. Numbers on curve in (B) indicate sample size at that DV location. (**F** and **G**) Dose/response map between total nuclear concentration of Dl and the low-diffusivity populations: correlated population (F) and uncorrelated population (G).

To test whether the relationships we observed also hold across intermediate levels of Dl and are not simply artifacts of measuring in two different fly lines, we leveraged the fact that the nuclear concentration of Dl varies with DV location. The natural gradient of Dl concentration, which is most intense at the ventral midline (DV coordinate of 0) and decays to near background levels roughly halfway around the embryo (DV coordinate of 0.5; Fig. 2A) (*14*, *19*), allows us to measure the effect of continuous variations in total nuclear Dl concentration within the same fly line. Therefore, we imaged different locations along the DV axis during mid-to-late nc 14, when the concentrations of the pools of Dl are each roughly at steady state (Fig. 1, B to E; see Materials and Methods). The longer duration of nc 14 allowed us to average the parameters obtained over many frames during which the parameters can be assumed to be at equilibrium, compared to other shorter ncs. We grouped embryos by their DV coordinate, with at least two embryos per bin (width of bin = 0.05).

As expected, we found that the total nuclear concentration of Dl decreases as the DV location moves away from the ventral midline (Fig. 2B). The total Dl concentration peaks at the ventral midline (~120 nM) and drops to below 50 nM on the lateral side (DV = 0.45). We noted that the free concentration of Dl stays roughly at a value of ~30 nM in all the DV locations (Fig. 2C). The slight variation observed is within noise and the free concentration stays unexpectedly constant (see the dashed line in Fig. 2C at the mean value) in contrast to what would be expected if it were a fraction of the total nuclear concentration. This concentration roughly agrees with the free concentrations we observed for the wt and reduced lines in the RICS time course (Fig. 1C).

The fraction of both pools of Dl composing the immobile fraction: The correlated fraction representing the DNA-bound pool determined by ccRICS (fig. S4A) and the uncorrelated fraction representing the pool immobilized likely due to the formation of clusters (fig. S4B) showed slight reduction with distance form ventral midline.

We found that the concentrations of both slowly moving pools of Dl decrease with increasing distance from the ventral midline due to the decrease in the total nuclear concentration of Dl (Fig. 2, D and E). This is expected, as a higher concentration of bound Dl is needed on the ventral side for driving the expression of target genes. Together with the nearly constant concentration of free Dl, we found no clear relationship between the concentrations of the slowly moving pools of Dl and the concentration of free Dl (fig. S4, C and D). In contrast, the relationship between the concentrations of these pools of Dl and the concentration of total Dl is almost linear (Fig. 2, F and G). Furthermore, a small extrapolation of these linear relationships reveals an *x*-intercept of ~30 nM of the total Dl concentration. Thus, our in vivo measurements along the DV axis further support that there exists a threshold of total Dl of ~30 nM that must be reached to facilitate the formation of the slowly moving populations of Dl (either by DNA binding or cluster formation), independent of the DV location. However, we cannot rule out that our observed relationship between total Dl and the slowly moving pools may be in the linear regime of a highly sigmoidal function.

### Threshold concentrations in Dl possibly indicate thermodynamic phase separation

The presence of a threshold concentration mentioned above is a phenomenon observed in LLPS (*48*). While this is a controversial topic, we asked whether any other hallmarks of LLPS exist in the Dl system. First, we noted that, in a pure-species system that undergoes phase separation, once a threshold concentration is reached, any increase in the total concentration changes relative volumes occupied by the phases, but not their concentrations (*48*). While this is only strictly true of a pure species system, it may be a reasonable approximation for the Dl system if other components do not strongly affect putative phase separation. If so, then the free population (representing the dispersed phase) and the slowly moving ones (both representing condensed phases) should each have constant concentrations. We observed a constant concentration of the dispersed phase, consistent with LLPS (Figs. 1C and 2C). On the other hand, our measurements of the condensed phase concentrations have been averaged over the whole nuclear volume. Under the assumption of LLPS, these averaged concentrations would be equal to the product of the actual (fixed) concentrations and the volume fraction of the respective condensed phases (see Materials and Methods). Furthermore, the volumes occupied by these phases, and thus our measured concentrations, would be directly proportional to the total nuclear concentration of Dl above the threshold of 30 nM, consistent with our observations (Fig. 2, F and G; see Materials and Methods).

Second, weak multivalent protein-protein interaction mediated by IDRs or low-complexity domains (LCDs) leads to LLPS (*5*, *48*–*51*). We thereby used predictive algorithms such as PSPredictor (*52*), PhaSePred (*53*), FuzDrop (*54*), and PSPHunter (*55*) to estimate the propensity of LLPS in case of Dl. All the algorithms returned a high score indicating that Dl is likely to phase separate (see table S1), as opposed to mNG, the fluorescent protein tagged to it, which shows very low propensity to phase separate. While the putative concentration threshold and IDRs intriguingly point to the potential of Dl to undergo LLPS, showing that an in vivo system in fact phase separates is difficult. Further in vivo measurements to infer the material properties of the clusters and the mechanism of formation (*48*, *51*) are needed before a definitive case can be made.

### SPT reveals slowly moving clusters of Dl

**Fig. 3.**
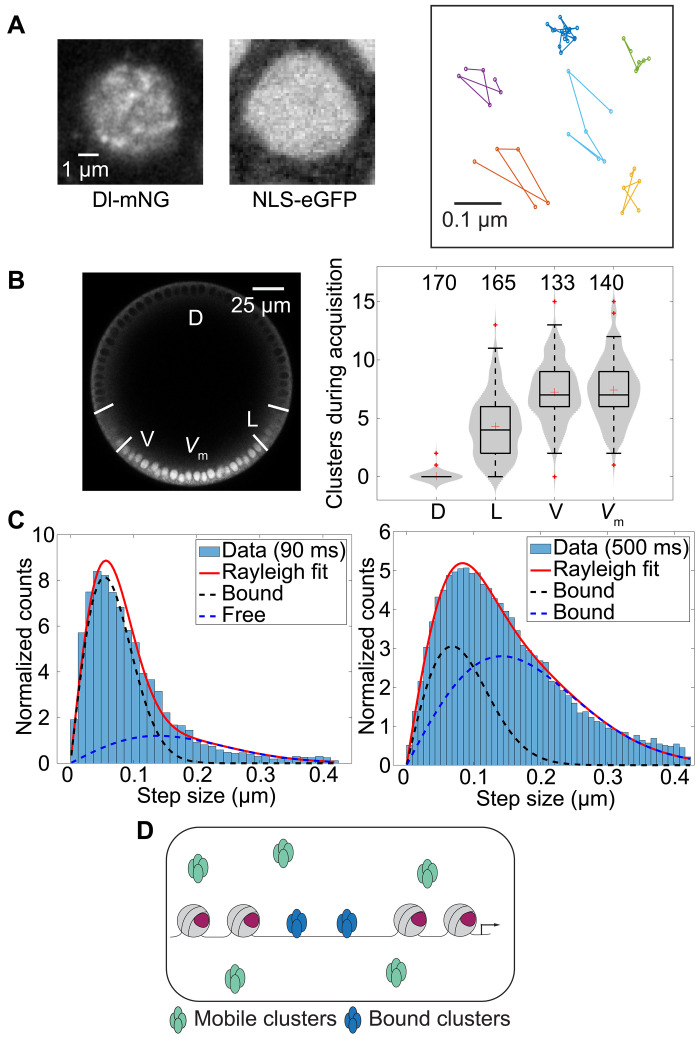
SPT to characterize Dl clusters. (**A**) Representative average image of a time series for Dl-mNG and nuclear localization signal (NLS)–eGFP and representative trajectories of the particles. (**B**) Number of clusters detected during acquisition on different DV locations as shown in the left panel. Numbers in the right panel indicate sample size. The image in the left panel is the same as that in the left panels of Figs. 1A and 2A for the ease of comparing the position of the region of interest in the DV axis across different experiments. (**C**) SSD and two-component model fitting for the determination of diffusivities and compositions of different populations for trajectories detected in 90-ms frame rate (left, *n* = 140 nuclei) and 500-ms frame rate (right, *n* = 118 nuclei). The dashed lines show the contribution of both the populations to the two-component Rayleigh fit. (**D**) Bound and slowly moving clusters are identified using SPT.

The results so far point to the presence of two pools of Dl with different diffusivities: fast (possibly free Dl) and slow (possibly clusters of Dl and/or DNA-bound Dl, corresponding to the uncorrelated and correlated populations, respectively) (Fig. 1A). Any clusters of Dl, either those known to be bound to DNA (*13*, *23*) or the putative clusters in the uncorrelated population, would be brighter than the single molecules of freely diffusing Dl. Therefore, we performed SPT using super-resolution imaging to identify and characterize the mobility of the clusters of Dl. We used the Zeiss Airyscan detector that allowed the fast imaging with an improved signal-to-noise ratio (SNR) compared to traditional confocal acquisitions, which is needed for particle tracking experiments (*56*). We imaged the ventral-most nuclei in the embryos at mid-nc 14 with a rapid frame rate (90 ms per frame). In the average image over the whole time series, bright puncta were clearly visible for Dl-mNG, whereas no such puncta were observed in control embryos expressing nuclear localization signal–enhanced green fluorescent protein (eGFP) (Fig. 3A). The Fiji Mosaic plugin was used to detect and track the particles (see Materials and Methods) (*57*), and we found clusters of Dl that lasted for several frames (Fig. 3A and movie S2). We found that the total number of particles detected during the acquisition time decreased with increasing distance from the ventral nuclei (Fig. 3B). This suggests that more clusters are formed at high levels of Dl. A trend similar to that was observed in case of the correlated and uncorrelated populations using RICS (Fig. 2, D and E).

To analyze the movement of these particles, we used a step size distribution (SSD) approach, in which the step sizes of particles between two consecutive frames are recorded. The shape of the distribution of these step sizes can be analyzed to determine not only the diffusivities of the particles but also whether multiple subpopulations with different diffusivities are present (*58*). Following this approach, we fitted a two-component Rayleigh distribution (see Materials and Methods) to the distribution of the step sizes (*33*) and found two populations with diffusion coefficients of 0.1 and 0.01 μm^2^/s (34 and 66% of the total clusters respectively; Fig. 3C). These diffusivities are very similar to those recently reported for DNA-bound hubs and for mobile (slowly moving) clusters, respectively, for the early *Drosophila* TFs Bcd and Cic (*33*). Hence, we interpret the slower population with 0.01 μm^2^/s diffusivity to be a DNA-bound population of Dl as the diffusivity is similar to that of His2Av or other DNA-bound TFs in *Drosophila* embryo (*10*) and the relatively faster population with 0.1 μm^2^/s diffusivity to be slowly moving clusters of Dl. To determine whether the two-component model is the best fit, we also fitted one- and three-component distribution models to our data. The one-component distribution model could not explain the data (see fig. S5A), and, in the three-component model, the first two components match those of the two-component model, while the third component is optimized to be 0% of the total population (fig. S5A). Notably, the population of free Dl [D ~3 to 4 μm^2^/s (*29*)] is not detected in this experiment because free Dl would not form bright clusters and because the frame time is too long to detect a free diffuser. This suggests that, at a long enough frame time, slowly moving clusters will be also blurred into the background and only the DNA-bound population will be detected (*10*, *59*). When we used a longer frame time of 500 ms, we could only detect the population of particles bound to DNA (Fig. 3C). The two-component Rayleigh model fit to the SSD gives diffusivities of 0.01 and 0.004 μm^2^/s representing 66 and 34% of the population, respectively. Although the diffusivity of 0.01 μm^2^/s is similar to that of the DNA-bound population found in the case shorter frame time of 90 ms, the other diffusivity suggests the presence of another bound population with even lower diffusivity. This implies that our “bound” or slowly moving populations are composed of complex subpopulations having varying diffusivities, as suggested previously (*10*).

Mean-squared displacement (MSD) (*60*) analysis, although only applicable for the determination of diffusivity when a single population is expected, can still provide qualitative information about the type of motion of the particles. The slope in the MSD versus Δ*t* plot and slope of the MSD/Δ*t* versus Δ*t* plot, where Δ*t* is the lag time, provide insights into the diffusivity regime of the particles. For the 500-ms frame time, we found the slope to be less than 1 in the MSD versus Δ*t* plot and negative in the MSD/Δ*t* versus Δ*t* plot (fig. S5B), indicating the presence of the population in the subdiffusion regime, which results from confined diffusion, consistent with movement of particles attached to the DNA (*10*, *57*, *61*, *62*). Together, these results suggest that there are at least two separate populations of clusters of Dl: DNA-bound and the slowly diffusing (Fig. 3D).

### Modified RICS imaging parameters simultaneously detect three populations

Our RICS experiments described in a previous section showed that there is a free population of Dl-mNG and a slowly moving population (*29*). Using SPT, we found that the slowly moving population is composed of at least two subpopulations: one bound to the DNA and another of slowly moving Dl clusters with a diffusivity of roughly D = 0.1 μm^2^/s. Hence, the SPT and RICS analyses together have detected a total of at least three different populations on the basis of diffusivity: freely diffusing Dl, slowly moving clusters, and DNA-bound Dl. However, the individual experiments could not detect all three populations together, as the free population detected in RICS moves too quickly to be detectable with SPT, while the slowly moving subpopulations cannot be readily distinguished in our previous RICS experiments.

**Fig. 4.**
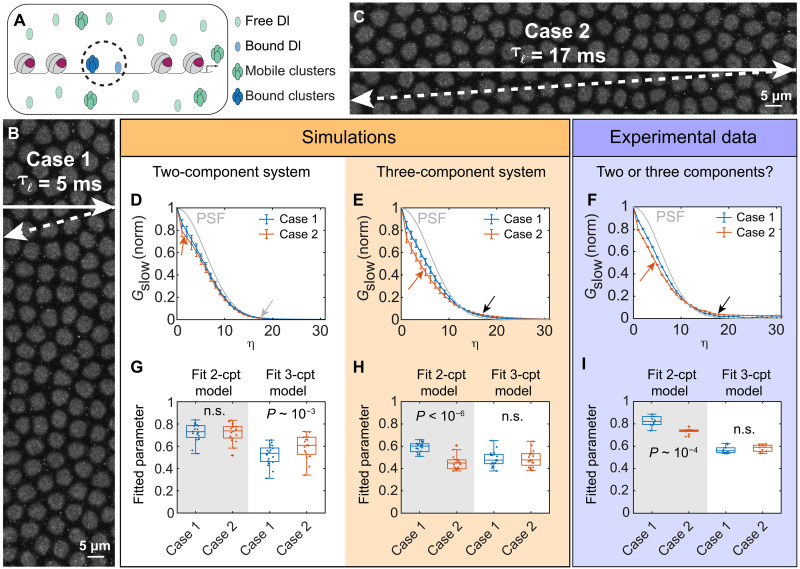
RICS experiments with varied line time to capture slowly moving clusters. (**A**) Populations identified; bound populations (circled) indistinguishable in this experiment. (**B** and **C**) Snapshots of ventral midline. Arrows denote laser path: horizontal arrow represents line scanning, dashed diagonal arrow represents line retracing; together, they roughly correspond to the line time. Case 1 (B) has a short line time (5 ms); case 2 (C) has the same embryo with the microscope axes reoriented by 90°, ensuring a longer line time (17 ms). (**D** and **E**) Slow-direction ACFs for a two-component (free Dl + immobile Dl) system (D) or three-component (free Dl + slowly moving clusters + immobile Dl) system (E) for cases 1 and 2; PSF shown in gray. Two-component system (D): The ACFs differ only slightly (red arrow) and neither ACF extends past the PSF (gray arrow). Three-component system (E): the ACFs visually differ (red arrow), and both ACFs extend past the PSF (black arrow). *n* = 20 for each Case. Error bars, SEM. (**F**) Experimental data. The average slow-direction ACFs visually differ (red arrow), and both extend past the PSF (black arrow). *n* = 9 embryos. Error bars, weighted SEM. (**G** and **H**) Model fits to the simulated two-component (G) or three-component (H) system. When the fitted model matches the underlying system [(G) left and (H) right], the values of the fitted parameter are indistinguishable between cases 1 and 2. However, mismatching the fitted model and the system [(G) right and (H) left] produces statistically different values of the fitted parameter between the two cases. (**I**) Two- and three-component models fitted to experimental data. The two-component model fits produced statistically different values of the fitted parameter between cases 1 and 2. However, fitting the three-component model produced statistically indistinguishable values of the fitted parameter between the two cases.

To simultaneously detect all three populations (Fig. 4A) and, in particular, to test the hypothesis that there is a slowly moving population of Dl distinguishable from truly immobile Dl, we used a longer line time for the RICS acquisitions. The longer line time would give the slowly moving clusters more time to diffuse to a new location during the raster scan, thus allowing us to detect them while still allowing us to detect the free Dl. Therefore, to alter the line time while holding as many other imaging parameters (such as pixel dwell time, image size, and frame time) as constant as possible, we imaged 4096 pixel–by–1024 pixel areas (133 μm by 33 μm) along the ventral midline of nine nc 14 embryos multiple times under two different cases (see Materials and Methods and movie S3). In case 1, we oriented the image acquisition vertically, such that the line time was $\tau_{\ell }∼5$ ms (Fig. 4B), consistent with the line time in our RICS acquisitions described in a previous section. In case 2, we flipped the orientation of the image acquisition, resulting in a line time of $\tau_{\ell }∼17$ ms (Fig. 4C), which should be able to detect particles with a diffusivity as low as roughly 0.1 μm^2^/s.

To determine the expected behavior of the system if slowly moving clusters are present, we simulated a two-component system (with a freely diffusing population and a truly immobile population, but no clusters), and a three-component system in which slowly moving clusters are present (see fig. S6, A and B). Our simulations showed that, if slowly moving clusters are not present, there would be very little difference in the slow direction cut of the autocorrelation function (ACF) between cases 1 and 2 (Fig. 4D, “Two-component system”; see also Materials and Methods), only a small deviation at low pixel shifts (see red arrow in Fig. 4D). In addition, the large-pixel-shift tails of the ACF should not extend past the point spread function (PSF) (see gray arrow in Fig. 4D). On the other hand, if there is also a third population of slowly diffusing clusters of Dl, there should be a clear difference in the slow direction cut of the ACF between cases 1 and 2 (compare blue curve to the red curve in Fig. 4E, “Three-component system”). Furthermore, the ACFs of both cases should extend beyond the tail of the PSF at large pixel shifts (black arrow in Fig. 4E). Our experimental results show that the slow direction of the ACF is clearly different between cases 1 and 2, and both extend past the PSF, suggesting there are three populations with different diffusivities present (Fig. 4F).

To quantitatively test our hypothesis that there is a slowly moving population of Dl, we fit two- and three-component models to our data. If we fit these models to the simulations of a two-component system, the fitted parameter of the two-component model (the fraction slowly moving) would not be statistically distinct between cases 1 and 2. However, the fitted parameter in the three-component model (the fraction of mobile clusters) would be statistically distinct between cases 1 and 2 (Fig. 4G) because the three-component model would not match the underlying two-component system. In contrast, if we fit these models to the simulations of a three-component system, the opposite would be true (Fig. 4H). In other words, if the model matches the system, fitted parameters should match between cases 1 and 2, while if the model is a mismatch to the system, the fitted parameters should also be mismatched between cases 1 and 2. When we fit the two models to our experimental data, we found a clear statistical difference between cases 1 and 2 for the two-component model fit, but no statistical difference between cases 1 and 2 for the three-component model fit (Fig. 4I). Therefore, the combination of RICS analyses for cases 1 and 2 clearly allow for simultaneous detection of all three populations of Dl, including the slowly moving clusters, which might be formed due to LLPS (Fig. 4A). Furthermore, our three-component model fits suggest that these slowly moving clusters have a weighting of $\phi_{1}=0.6$. These results further verify our observations for the spatiotemporal variation using RICS and the SPT results.

## DISCUSSION

**Fig. 5.**
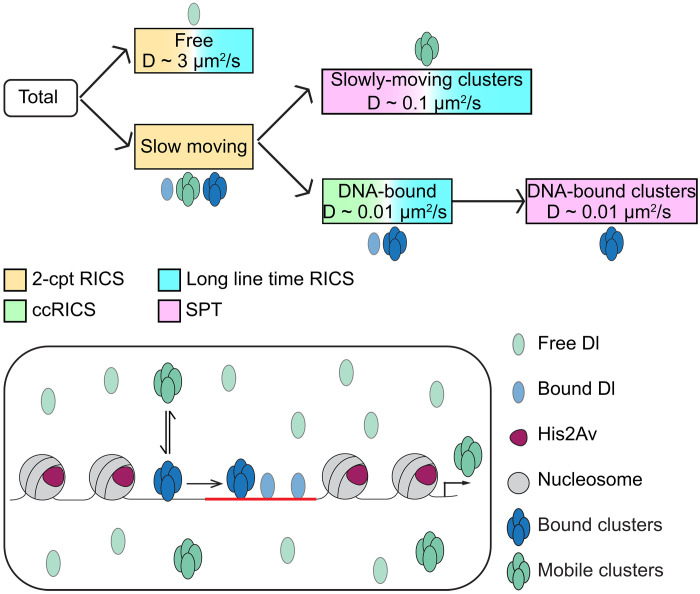
Model of Dl-DNA binding as indicated by multiple experimental techniques. The total population of Dl in the nuclei is composed of free and slow populations as indicated by RICS. The slow population is composed of DNA-bound population (indicated by ccRICS) and slowly moving clusters. SPT can differentiate between the DNA-bound clusters and slowly moving clusters. Last, long line time RICS can detect the presence of all the constituent populations together. Both the experimental techniques and the different populations are color coded.

Despite decades of research, the molecular mechanisms behind the precise gene regulation by sequence-specific TFs, particularly mechanisms linking nuclear concentration of the TF to its DNA binding (a crucial step in gene regulation), remain unresolved (*22*, *63*). Transcriptional regulation is dependent on TF dynamics such as the total concentration, fraction bound, and hub formation (*5*). Therefore, in this study, we investigated how the bulk concentration of Dl relates to DNA binding and hub formation. Using experiments that span multiple length and timescales, we detected at least three different populations of Dl having distinct diffusivities present in the nuclei: freely diffusing (D ~ 3 μm^2^), previously unobserved slowly moving clusters (D ~ 0.1 μm^2^), and a DNA-bound population (D ~ 0.01 μm^2^; see Fig. 5).

Recent work has suggested that clusters, or hubs, of TFs are crucial in DNA binding. For example, DNA-bound hubs of the morphogen Bcd have been proposed recently to facilitate the transcriptional activity by locally increasing the concentration (*10*, *11*) or by acting as fast sensor of concentration (*64*). Similarly, enrichment of puncta of Dl near individual transcription sites has been observed (*13*, *23*). In addition to DNA-bound hubs, a recent study has shown that Bcd and Cic, two TFs active in the early *Drosophila* embryo when Dl is also active, form slowly moving aggregates in the nuclei (D ~ 0.1 μm^2^/s) (*33*). In this work, we found a similar, previously unobserved population of slowly moving clusters of Dl, suggesting that this might be a common mechanism of transcriptional regulation by morphogen gradients. As the presence of mobile clusters of TF has only recently been found, their functional role is unclear, and while it has been suggested that these mobile clusters may provide an efficient search mechanism for the target sites (*33*), further characterization is needed. Thus, gene regulation models in eukaryotes must take the formation of DNA-bound as well as slowly moving hubs of TFs into account.

In addition to identifying three subpopulations of Dl, we also leveraged the variation of Dl concentration across ncs 11 to 14, along the DV axis, and in two different fly lines to map the relationships among them. Strikingly, we found that the free concentration of Dl remained roughly the same (~30 nM), regardless of the bulk concentration, indicating the possibility of a threshold concentration of total Dl, above which further increases in Dl became exclusively allocated to the slowly moving populations. Further supporting the presence of a threshold in bulk concentration, the dose/response maps connecting the two slowly moving populations to the total Dl concentration were linear, extrapolating back to this same 30 nM threshold in total Dl concentration. Thus, our nucleus-level measurements, while not as specific as those that focus on individual enhancers (*13*), have provided crucial details regarding the mechanisms of DNA binding and cluster formation.

The presence of a threshold in the total nuclear concentration of Dl, above which no further free Dl is allocated, is a hallmark LLPS of biomolecular condensates (*65*). While the clusters of Dl forming from LLPS is an intriguing possibility, other work suggests the opposite, and overall, LLPS of TFs has been a hotly debated topic in the past decade (*50*, *66*–*68*). LLPS has been proposed to play a role in buffering biological fluctuations, aiding in robustness of certain pathways, or repressing pathways whose functioning requires noise (*50*, *69*). However, quantitative methods to provide definitive evidence of LLPS are still lacking in the field [reviewed in (*51*)]. Therefore, regardless of the mechanistic status of Dl cluster formation, our in vivo quantitative measurements and methodologies provide a general, quantitative framework for testing properties of clusters for LLPS-like behavior by the detecting relationships among the constituent populations in vivo.

While subpopulations detected in one experiment can be similar to those detected in other experiments, we acknowledge these similar subpopulations may not necessarily be identical across experiments. For example, the correlated and uncorrelated populations from RICS/ccRICS experiments may not exactly map one-to-one onto the DNA-bound clusters (0.01 μm^2^/s) and slowly moving clusters (0.1 μm^2^/s) detected in the SPT experiments, as some of the correlated RICS population could be DNA-bound monomers or dimers of Dl. In addition, we acknowledge the possibility that there may be populations having intermediate composition and diffusivity not identified in our experiments. One further caveat is that clusters of fluorescently labeled molecules, by definition, have a higher molecular brightness than the monomers and dimers, which affect the quantitative values of the RICS analyses (*27*, *36*, *70*). In particular, corrections for differing brightness values would result in a slight increase to the total concentration of Dl, and the concentration of the other subpopulations would also scale accordingly. Even so, our general findings, including the discovery of mobile clusters of Dl and the linear/threshold relationship of slowly moving populations to the total Dl concentration, are not affected by these caveats. Last, although the threshold-based formation of the bound populations can be explained by LLPS, we acknowledge that affinity-based binding model can still explain some of our findings indicating that both the phenomena might drive Dl binding and thereby gene regulation.

The existence of clusters, both DNA-bound and mobile, have profound implications for our understanding of gene regulation. Put within the broader context, these clusters must be accounted for along with other crucial parameters that explain binding of a TF, such as the chromatin accessibility and cooperativity with pioneer-like factors. Together, our results support a model in which, during the early ncs, owing to its lower concentration, Dl binds only the accessible sites created by pioneer factors. During nc 14, when the nuclear concentration of Dl is high, binding is driven by the concentration of Dl, perhaps by clusters of Dl. This is in agreement with the proposed cooperativity between the pioneer factor Zld and Dl and the sporadic/delayed expression of Dl target genes in Zld mutant embryos (*41*, *71*–*73*). Our results also suggest that, during nc 14, binding occurs when a threshold concentration of free Dl is exceeded. Hence, different populations of Dl at varying concentrations within and across nuclei must be taken into account to understand its concentration-dependent transcriptional interpretation. More broadly, we anticipate that similar detailed measurements of nuclear subpopulations will be needed for other TF systems, potentially pointing to generalizable rules of transcriptional regulation (*10*, *11*, *13*, *33*, *36*, *64*, *74*).

## MATERIALS AND METHODS

### *Drosophila* strains

The fly strain used for all the imaging for Dl is *yw/w*; *dorsal-mNeonGreen/cyo*; + unless otherwise stated. For the reduced level of Dl we used, *yw/w*; *dorsal-mNeonGreen-dsRed/cyo*; +. The strain used for observing the populations in two copies of Dl-mNeonGreen is *yw/w*; *dorsal-mNeonGreen/dorsal-mNeonGreen*; +. The other fly strains used are: w[*]; *P{w[+mC] = His2Av-mRFP1}II.2* [Bloomington Drosophila Stock Center (BDSC) #23651] and w[*]; *P{w[+mC] = His2Av-mRFP1}III.1* (BDSC #23650) for nuclear segmentation and ccRICS using His2Av-RFP, w[*]; *P{w[+mC] = His2Av-EGFP.C}2/SM6a* (BDSC #24163) for using His2Av-eGFP as photobleaching control in the particle tracking experiments.

### *Drosophila* husbandry and sample preparation

The fly stocks were grown on standard cornmeal-molasses-yeast medium at 25°C. Fly cages were set with males and females of desired fly lines 2 days before imaging and kept at room temperature. Grape juice plates were streaked with yeast paste and placed on the bottoms of the cages for egg laying. The plates were changed once every day. On the day of imaging, the grape juice plates were placed for oviposition for an hour after which it was removed to collect the embryos. The embryos were washed from the plate into a mesh basket using deionized water. They were then dechorionated in bleach for 30 s and washed again with deionized water to remove residual bleach. The embryos were mounted in 1% solution of low melting point agarose (IBI Scientific, IB70051) in deionized water on a glass-bottom petri dish (MatTek, P35G-1.5-20-C). The mounting was done in such a way that the ventral surface of the embryo touched the coverslip in the petri dish for imaging the ventral nuclei. For imaging other DV positions, the angle at which the embryo touched the coverslip was changed by rotating it slightly with a hair loop. For imaging the spatial gradient, the embryo at early nc 14 was mounted end-on with the anterior pole touching the coverslip. The agarose was let to solidify after which deionized water was poured in the dish to avoid drying of the agarose covering the samples.

### Spatial and temporal variation RICS

All the imaging was performed using LSM 900 (Carl Zeiss, Germany) confocal laser scanning microscope. For the confocal imaging C-Apochromat 40×/1.2 water immersion Korr objective, 488-nm laser for the mNeonGreen, 561-nm laser for RFP, and GaAsP-PMT detector were used. Embryo stage was estimated on the basis of the His2Av-RFP signal. Image acquisition was started when the embryos reached the desired stage: nc 10 for the temporal variation study and mid nc 14 for the spatial variation study. A frame size of 1024 pixels by 1024 pixels at 5× zoom was used. This corresponded to a pixel size of 31.95 nm and a pixel dwell time of 2.06 μs at a frame time of 5.06 s. The acquisition was continued until the embryo gastrulated as indicated by the nuclear morphology.

To minimize photobleaching, the ROI was moved to a different part of the embryo after every 12 frames ensuring that all the locations are aligned in the same DV coordinate. RICS analysis on the 12 frame acquisitions of time series enabled the precise determination of the temporal dynamics of the total nuclear concentration and binding of Dl.

### Imaging and quantifying the Dl gradient

The embryo cross section was imaged at a location of 150 μm from the anterior pole from early nc 14 until gastrulation. This was done for both reduced and wt embryos. The nuclear fluorescence intensities along the Dl gradient were extracted following established protocols (*14*). The intensities of each embryo during the period of −26 to −20 min from gastrulation were averaged. These averaged values for multiple embryos of the same type (reduced or wt) were again averaged to get the intensities along the Dl gradient. A Gaussian was then fitted to the averaged intensities.

### RICS analysis and fitting of ACF models

We performed RICS analysis according to previously published protocols (*29*, *36*). In brief, two-dimensional (2D) RICS ACFs were built from data by the following formula

$$G_{j}\left(\Delta x,\Delta y\right)=\frac{\langle I_{j}\left(x,y\right)I_{j}\left(x+\Delta x,y+\Delta y\right)\rangle }{{\langle I_{j}\left(x,y\right)\rangle }^{2}}-1$$ (1)

where I is the intensity of the image, (x,y) refers to the spatial coordinates within the image, the “*j*” subscript refers to frame “*j*” of the time series, and the angle brackets denote ensemble average in both x and y. In building the ACF, we used only pixels corresponding to the nuclei, determined by segmentation of the His2Av-RFP channel. The functions $G_{j}\left(\Delta x,\Delta y\right)$, for each frame, were built using a fast Fourier transform protocol in Matlab, and these ACFs were averaged together for all frames in a given group. The result was a time series of 2D ACFs, each of which corresponded to a given grouping of 7 to 12 frames. Background subtraction was performed on the fly by examining the histogram of intensities and fitting a Gaussian to the lowest intensity pixels in the image (more details below in “Background subtraction” section).

The theoretical 2D RICS ACF, G(Δx,Δy), is the following

$$\begin{array}{c} G\left(\Delta x,\Delta y\right)=\\ A\left(\frac{1}{1+\frac{4D\left(\tau_{p}\Delta x+\tau_{\ell }\Delta y\right)}{w_{0}^{2}}}\right)\sqrt{\frac{1}{1+\frac{4D\left(\tau_{p}\Delta x+\tau_{\ell }\Delta y\right)}{w_{z}^{2}}}}\text{exp}\left(-\frac{\left({\Delta x}^{2}+{\Delta y}^{2}\right)\Delta r^{2}}{w_{0}^{2}+4D\left(\tau_{p}\Delta x+\tau_{\ell }\Delta y\right)}\right) \end{array}$$ (2)

where A is the amplitude; D is the diffusivity; $w_{0}$ and $w_{z}$ are the radii of the PSF in the xy plane and the axial (z) direction, respectively; Δr is the *xy* size of a pixel; $\tau_{p}$ and $\tau_{\ell }$ are the pixel dwell time (determined by the scan speed) and line time (determined by a combination of the scan speed and number of pixels in the width of the image), respectively; and Δx and Δy are the pixel shifts in the fast and slow directions, respectively.

In practice, $A=\gamma /\left(V_{\mathrm{PSF}}\bar{c}\right)$, where $\gamma =\sqrt{2}/4$ is a factor that accounts for the uneven illumination airy unit, $\bar{c}$ is the average concentration in the confocal volume, and $V_{\text{PSF}}=\pi^{\frac{3}{2}}w_{0}^{2}w_{z}$ is the effective volume of the PSF. The fast direction of the data-derived 2D ACF, at each time point, was used to fit the fast direction cut of the theoretical 2D ACF, G(Δx,0), which reduces to a Gaussian equation that approximates the microscope’s PSF

$$G\left(\Delta x,0\right)\approx \left(A-B\right)\text{exp}\left(-\frac{{\Delta x}^{2}\Delta r^{2}}{w_{0}^{2}}\right)+B$$ (3)

To improve the robustness of the fit, a small, adjustable background constant, B, was added and $w_{0}$ was allowed to vary slightly. The parameter B was constrained to have a magnitude less than 10^−3^. This first fitting step gave us an estimate of the ACF amplitude, A, and hence, the average total concentration.

After obtaining an estimate of A, we held it fixed and used the entire 2D ACF to fit $G_{2c}(\Delta x,\Delta y)$, which is a linear combination of two components, a freely diffusing population and a slowly moving population (*29*)

$$G_{2c}\left(\Delta x,\Delta y\right)=\left(A-B\right)\left[\phi G_{0}\left(\Delta x,\Delta y\right)+\left(1-\phi \right)G_{1}\left(\Delta x,\Delta y\right)\right]+B$$ (4)

where $G_{0}\left(\Delta x,\Delta y\right)$ is given by Eq. 2 with A=1 and D=0, $G_{1}\left(\Delta x,\Delta y\right)$ is given by Eq. 2 with A=1 and D nonzero, and the linear combination weight, ϕ, is the fraction slowly moving. Note that the parameter B in Eq. 4 may have a different value from the one found in Eq. 3. To avoid overfitting, we assumed D=3 μm^2^/s.

### Background subtraction

The amplifier offset value on the LSM 900 confocal was set to result in a zero-current intensity of roughly 100 arbitrary units (AU). To account for this, each RICS time course image stack was background subtracted to align the zero intensity with the zero-current intensity in the following manner. First, the histogram of intensities of the first time frame was generated. Next, a Gaussian was fit to the lowest intensity pixels in the image. The mean and SD of the Gaussian fit were approximately 100 and 10 AU, respectively. Next, this mean value was subtracted from the image intensity at each pixel and time frame.

Additional background levels of intensity could plausibly arise from autofluorescence of the embryo tissue. To control for this, we imaged embryos from *w; His2Av-mRFP1/Cyo; PrDr/TM3* parents (lacking Dl-mNG) using the same microscope settings as those for acquiring the time courses for RICS analysis. The fluorescence intensity observed in the green channel in these embryos lacking Dl-mNG represents the background fluorescence level. The presence of this background intensity will change the estimated concentrations for all the populations (total, free, correlated, and uncorrelated) derived from the RICS analysis by a factor of (*I* − *I*_0_)^2^/*I*^2^, where *I* and *I*_0_ are the Dl-mNG nuclear intensity and the background intensity respectively. We found that the obtained background intensities were roughly 1 to 2% of that of Dl-mNG during ncs 12 to 14 implying a 4% correction to the concentration at most, which can be ignored safely.

### Fitting ccRICS CCFs to estimate correlated binding

We performed analysis of ccRICS according to previously published work (*29*, *36*). In brief, the 2D cross-correlation function (CCF) between Dl-mNG and His2Av-RFP was computed through a fast Fourier transform protocol in Matlab. This 2D CCF was then used to fit a zero-diffusion 2D model of cross correlation (*29*)

$$G_{\mathrm{cc}}\left(\Delta x,\Delta y\right)=\left(A_{\mathrm{cc}}-B\right)\text{exp}\left(-\frac{{\left(\Delta x\Delta r-d_{x}\right)}^{2}+{\left(\Delta y\Delta r-d_{y}\right)}^{2}}{{\hat{w}}_{0}^{2}}-\frac{d_{z}^{2}}{{\hat{w}}_{z}^{2}}\right)+B$$ (5)

where $d_{x},d_{y},d_{z}$ represent the displacements between the centers of the two PSFs in the *x*, *y*, and *z* directions, respectively; ${\hat{w}}_{0}$ and ${\hat{w}}_{z}$ are the average PSF sizes for the planar (*xy*) and axial (*z*) directions, respectively; $A_{\text{cc}}$ is the amplitude of the CCF; Δr is the pixel size (see Eq. 2); and B is a background parameter (see Eqs. 3 and 4). The axial displacement was previously estimated by imaging fluorescent beads (*36*) and the factor $\text{exp}\left(d_{z}^{2}/{\hat{w}}_{z}^{2}\right)$ was found to be roughly equal to 1.02, implying that the factor could be safely ignored. The average PSF sizes are defined as ${\hat{w}}_{0}^{2}=0.5\left(w_{0,g}^{2}+w_{0,r}^{2}\right)$ and ${\hat{w}}_{z}^{2}=0.5\left(w_{z,g}^{2}+w_{z,r}^{2}\right)$, and the subscripts g and r denote the green and red channels, respectively. The fraction of Dl-mNG correlated to His2Av-RFP (the correlated fraction) is related to the ratio of $A_{\text{cc}}$ and the ACF amplitude of the red channel, $A_{\text{red}}$

$$\text{correlated fraction}=\frac{{\hat{w}}_{0}^{2}{\hat{w}}_{z}}{w_{0,r}^{2}w_{z,r}}\frac{A_{\text{cc}}}{A_{\text{red}}}$$ (6)

### Relationship between total concentration and condensed phase

Under the assumption of LLPS, to derive the relationship between free Dl (putative dispersed phase) and the correlated and uncorrelated pools of the slowly moving population (putative condensed phases 1 and 2, respectively), we used the pure species behavior as the foundation. In this case, all phases in equilibrium would have a fixed concentration with respect to varying total concentration, and variations in total concentration would change the volume partitioning among the two phases. We define ${\bar{C}}_{0}$, ${\bar{C}}_{1}$, and ${\bar{C}}_{2}$ as the fixed concentrations of the dispersed phase and the two condensed phases, respectively. We further define $V_{0}$, $V_{1}$, and $V_{2}$ as the volumes occupied by the dispersed phase and the two condensed phases, respectively, and note that the total volume of the nucleoplasm available to Dl is $V_{\text{tot}}=V_{0}+V_{1}+V_{2}$.

As our measurements of the free, correlated, and uncorrelated concentrations ($C_{\text{meas},0}$, $C_{\text{meas},1}$, and $C_{\text{meas},2}$, respectively) are averages over the entire volume, $V_{\text{tot}}$, they relate to their respective fixed concentrations by

$$C_{\text{meas},0}={\bar{C}}_{0}V_{0}/V_{\text{tot}}$$ (7)

$$C_{\text{meas},1}={\bar{C}}_{1}V_{1}/V_{\text{tot}}$$ (8)

$$C_{\text{meas},2}={\bar{C}}_{2}V_{2}/V_{\text{tot}}$$ (9)

respectively. If $V_{1}+V_{2}\ll V_{0}$, then Eq. 7 reduces to $C_{\text{meas},0}={\bar{C}}_{0}$, which explains how our measurements of free Dl remain roughly fixed (at 30 nM) with varying total Dl concentration.

The total concentration relates to the phase volumes by

$$C_{\text{tot}}=C_{\text{meas},0}+C_{\text{meas},1}+C_{\text{meas},2}=\frac{{\bar{C}}_{0}V_{0}+{\bar{C}}_{1}V_{1}+{\bar{C}}_{2}V_{2}}{V_{\text{tot}}}$$ (10)

where, in the second line, we have used Eqs. 7 to 9. Under the continued assumption that $V_{1}+V_{2}\ll V_{0}$, Eq. 10 can be reduced to

$$C_{\text{tot}}\approx {\bar{C}}_{0}+\frac{{\bar{C}}_{1}V_{1}}{V_{\text{tot}}}+\frac{{\bar{C}}_{2}V_{2}}{V_{\text{tot}}}$$ (11)

As the three phase concentrations and $V_{\text{tot}}$ are constant, Eq. 11 implies that $V_{1}$ and $V_{2}$, and thus also $C_{\text{meas},1}$ and $C_{\text{meas},2}$, are in a direct linear relationship with the difference between the total concentration, $C_{\text{tot}}$, and the concentration threshold, ${\bar{C}}_{0}$.

### Varying line time experiments

In the experiments with varied line times, the pixel size and pixel dwell time were kept the same as the previous experiments at 31.95 nm and 2.06 μs, respectively. The line time was varied by changing the frame size. For the shorter line time (case 1), the frame size was 1024 pixels by 4096 pixels, resulting in a line time of 5 ms. For the same embryo, the longer line time (case 2) was obtained by rotating the ROI by 90° (exchanging the *x* and *y* directions of the image), resulting in a frame size of 4096 pixels by 1024 pixels and a line time of 17 ms. This ensured that the imaging parameters will be conserved and each embryo will be imaged with two different line times, the short and the long one, at the exact same DV position. Thus, with the same number of pixels, pixel size, and pixel dwell time the images with frame size of 4096 pixels by 1024 pixels have a line time just under four times longer than that of the images with 1024 by 4096 for the same DV location. To minimize and account for photobleaching, the ROI was rotated after 12 frames and for some of the embryos the longer line time experiments were performed first and then the shorter line time ones whereas for the other embryos the order was reversed.

### Simulations of varying line time experiments

We simulated the ACFs of both cases (the short line time and long line time) for two different systems. The first system was a two-component system with freely diffusing Dl-mNG and DNA-bound Dl-mNG (zero diffusion), simulated using Eq. 4. We set the baseline diffusivity of free Dl to be 3 μm^2^/s and the baseline slowly moving fraction (ϕ) to be 0.75.

The second system was a three-component system with the extra component being a slowly moving cluster of Dl-mNG. In this system, we assumed that the slowly moving fraction, set at 0.75 for the two-component system, was split into a baseline of $\phi_{0}=0.25$ for the fraction bound to DNA and a baseline of $\phi_{1}=0.50$ for the fraction of slowly moving clusters, consistent with our experimental measurements of the correlated and uncorrelated fractions (see fig. S2, A and B)

$$\begin{array}{c} G_{3c}\left(\Delta x,\Delta y\right)=\\ \left(A-B\right)\left[\phi_{0}G_{0}\left(\Delta x,\Delta y\right)+\phi_{1}G_{1}\left(\Delta x,\Delta y\right)+\left(1-\phi_{0}-\phi_{1}\right)G_{2}\left(\Delta x,\Delta y\right)\right] \end{array}$$ (12)

where $G_{0},G_{1},G_{2}$ are each given by Eq. 2 with A=1 and with baseline diffusivities of 0, 0.1, and 3 μm^2^/s, respectively.

We ran 20 simulations of each system with 10% random noise added to the parameters. After the simulated ACFs were constructed according to Eq. 4 (two-component system) or Eq. 12 (three-component system), we added 2% Gaussian noise to the ACFs simulate experimental error. The smoothness of the resulting ACFs were comparable by-eye to the smoothness of our typical experimental ACFs. The means of the 20 simulations are found in Fig. 4 (C and D), with error bars being the SD.

We fit each of these simulations to either a two-component model (Eq. 4) or a three-component model (Eq. 12). To avoid overfitting, we allowed only one fitted parameter in each case: ϕ for the two-component model and $\phi_{1}$ for the three-component model. In both cases, we fixed the diffusivity of free Dl-mNG to be 3 μm^2^/s (*29*). For the three-component model, we additionally fixed $\phi_{0}$ to be 0.25 and the diffusivity of slowly moving clusters to be 0.1 μm^2^/s.

### RICS analysis of varying line time experiments and fitting of ACF models

The RICS analysis of our time course data for cases 1 and 2 in the varying line time experiments was performed as described above. Once the data-derived ACFs were computed, they were used to fit a two-component model (Eq. 4) and a three-component model (Eq. 12). As described above, to avoid overfitting, we held fixed all parameters except for ϕ for the two-component model and $\phi_{1}$ for the three-component model, and we fixed the diffusivity of free Dl-mNG to be 3 μm^2^/s and the diffusivity of slowly moving clusters to be 0.1 μm^2^/s. We fixed $\phi_{0}$ to be equal to the value of correlated fraction for that time course.

### Super-resolution imaging

The particle tracking experiments require rapid frame rates and high spatial resolution, and hence, we used the fast Airyscan detector for improved spatial resolution and SNR (*56*). All the images were taken on the ventral side of the embryos at mid-nc 14. Plan-Apochromat 63×/1.4 oil differential interference contrast M27 objective and 488-nm excitation laser for the Dl-mNeonGreen were used. All the images have a pixel size of 42 nm. For the SSD experiments, the rapid frame rate of 87 ms was obtained by having a frame size of 167 pixels by 167 pixels and the time series consisted of 35 frames. Whereas the longer duration of 500 ms was obtained by having a frame size of 380 pixels by 380 pixels and the time series consisted of 50 frames to detect the DNA-bound clusters that last for a longer time.

### Particle detection and tracking

The Fiji Mosaic plugin was used for particle detection and tracking (*57*), using the following parameters: particle radius, 6 pixels; cutoff score, 0%; and intensity percentile, 0.22%. For particle linking, the maximum step length was set at 10 pixels and the link range 1 frame. Any trajectory lasting less than three steps was discarded.

### Localization precision

To determine the localization precision, we imaged immobilized fluorescent beads (*33*, *75*, *76*). The beads (Fluoresbrite YO Carboxylate Microspheres 0.20 μm, Polysciences Inc., catalog no. 19391-10) were washed twice with deionized water and then mounted in low melting point agarose on a glass-bottom petri dish in the same way as the embryos to emulate the experimental conditions. The imaging was then performed using the same microscope parameters as those used for the embryos for the 87-ms frame rate. Since localization precision depends on *SNR* (*77*, *78*), which in turn depends on laser power, we used eight different laser power levels for the imaging: 0.025, 0.05, 0.075, 0.1, 0.2, 0.3, 0.5, and 0.8%. For each laser power, 10 different time series having 35 frames each at 10 different locations were acquired. The mean diffusivity was calculated using the MSD method (*33*, *75*) instead of the SSD method since the beads are immobilized and thus expected to have long-term trajectories, and we do not expect multiple populations with different diffusivities. We calculated the localization precision for all the acquisitions from the diffusivity ($\sigma^{2}=4D∆t$) obtained from the Fiji Mosaic plugin (*33*, *57*, *75*). The localization precision as a function of *SNR* was then empirically fit to the following equation (*57*, *78*)

$$\sigma^{2}=\frac{A}{\mathrm{SNR}}+\frac{B}{\mathrm{SN}R^{2}}$$ (13)

where the first term is due to background noise and the second term is due to shot noise. *SNR* is defined as the ratio of the mean intensity of the signal and SD of the background. The constants *A* and *B* were determined by fitting Eq. 13 to the data in the plot of $\sigma^{2}$ versus *SNR* plot using lsqcurvefit solver in Matlab (fig. S5C). The mean intensity of the signal in the embryos was determined at the coordinates of detected particles obtained from the Mosaic plugin and the SD of the intensity outside the detected particles inside the nucleus was determined. The ratio gives the *SNR* for our samples. The localization precision was then calculated using Eq. 13 at the determined SNR.

### Step size distribution

The possibility that multiple populations with different diffusivities might be present and the presence of trajectories exhibiting short-term motion led us to use the SSD for the estimation of diffusivities. The length of the steps taken between consecutive frames were calculated and the SSD of the particles was obtained. The probability density function of the step sizes was fit with a two-component Rayleigh mixture model (*10*, *33*, *60*, *79*)

$$P\;\left(r,∆t\right)=\sum_{i=1}^{k}f_{i}\frac{r}{2\left(D_{i}∆t+\sigma^{2}\right)}\text{exp}\left(-\frac{r^{2}}{4\left(D_{i}∆t+\sigma^{2}\right)}\right)$$ (14)

where $f_{i}$ is the fraction of the population *i* (and thereby $\sum_{i=1}^{k}f_{i}=1$), $D_{i}$ is the diffusivity of the population *i*, ∆t is the time between the steps, the localization precision (from the immobilized fluorescent beads experiments) is $\sigma^{2}$ = $4.5\times 10^{-4}\;\mu m^{2}$, and *r* is the step size. Since the imaging dataset is a 2D time course, it is plausible that some of the particle having higher diffusivity will move out of focus during imaging, resulting in an under sampling of the population with higher diffusivity thereby an over sampling of the population with lower diffusivity (*59*, *60*). This must be accounted for in the case of single-molecule tracking that detects free population having very high diffusivities in addition to the slow populations. However, this will likely have a small effect on our results since we are detecting only the particles either moving-slowly or bound to the DNA, in both cases having comparatively lower diffusivity than a free population. The correction can be safely overlooked, and Eq. 14 can be used for the long frame time of 500 ms as we only detect the DNA-bound populations in this case. However, for the case of shorter frame time of 90 ms, we added a correction factor $Z_{\text{corr}}$, which is a measure of fraction of particles having higher diffusivity remaining at time delay ∆t, in Eq. 14 to account for the defocalization bias. The modified equation becomes

$$\begin{array}{c} P\;\left(r,∆t\right)=\frac{F_{\text{bound}}}{F_{\text{bound}}+Z_{\text{corr}}\left(∆t\right)F_{\text{free}}}\frac{r}{2\left(D_{\text{bound}}∆t+\sigma^{2}\right)}\text{exp}\left(-\frac{r^{2}}{4\left(D_{\text{bound}}∆t+\sigma^{2}\right)}\right)+\\ \frac{Z_{\text{corr}}\left(∆t\right)F_{\text{free}}}{F_{\text{bound}}+Z_{\text{corr}}\left(∆t\right)F_{\text{free}}}\frac{r}{2\left(D_{\text{free}}∆t+\sigma^{2}\right)}\text{exp}\left(-\frac{r^{2}}{4\left(D_{\text{free}}∆t+\sigma^{2}\right)}\right) \end{array}$$ (15)

where, $F_{\text{bound}}+F_{\text{free}}=1$, and the normalization of the equation by the factor $F_{\text{bound}}+Z_{\text{corr}}F_{\text{free}}$ ensures that the sum of the weights of both the components in the two-component Rayleigh fit is also equal to 1

$$\begin{array}{c} Z_{\text{corr}}\left(∆t\right)=\\ \frac{1}{∆z}\int_{-\frac{∆z}{2}}^{\frac{∆z}{2}}\left\{1-\sum_{n=0}^{\infty }{\left(-1\right)}^{n}\left[\text{erfc}\left(\frac{\frac{\left(2n+1\right)∆z}{2}-z}{\sqrt{4D_{\text{free}}∆t}}\right)+\text{erfc}\left(\frac{\frac{\left(2n+1\right)∆z}{2}+z}{\sqrt{4D_{\text{free}}∆t}}\right)\right]\right\}\mathrm{dz} \end{array}$$ (16)

Here, Δ*z* is equivalent to axial detection range, which is ~0.7 nm for the 63×/1.4 numerical aperture objective used for our experiments. However, Eq. 16 is based on an absorbing boundary condition that assumes that any particle touching or moving past the boundary are lost. This effect has been corrected by considering that the actual ∆z should be larger than the axial detection range and the empirical relation has been determined to be $∆z=\text{axial detection range}+0.21\sqrt{D_{\text{free}}}$ (*79*). We changed the number of components to 1 and 3 (fig. S5A) to justify the use of two-component model. The values of diffusivities and fraction of the population in Eqs. 14 and 15 were estimated using the Matlab optimization function fmincon.
