# Supplementary material for: Global maps of transcription factor properties reveal threshold-based formation of DNA-bound and mobile clusters
Source: Sci Adv. 2026 Feb 27;12(9):eady3909. doi: 10.1126/sciadv.ady3909 (PMC12947891; doi:10.1126/sciadv.ady3909)
Supplement: Supplementary file 1 — Figs. S1 to S6 Table S1 Legends for movies S1 to S3 [file sciadv.ady3909_sm.pdf]

Science Advances
AAAS

Supplementary Materials for

Global maps of transcription factor properties reveal threshold-based formation of DNA-bound and mobile clusters

Sadia Siddika Dima *et al.*

Corresponding author: Gregory T. Reeves, gtreeves@tamu.edu

Sci. Adv. **12**, eady3909 (2026)
DOI: 10.1126/sciadv.ady3909

The PDF file includes:

Figs. S1 to S6
Table S1
Legends for movies S1 to S3

Other Supplementary Material for this manuscript includes the following:

Movies S1 to S3

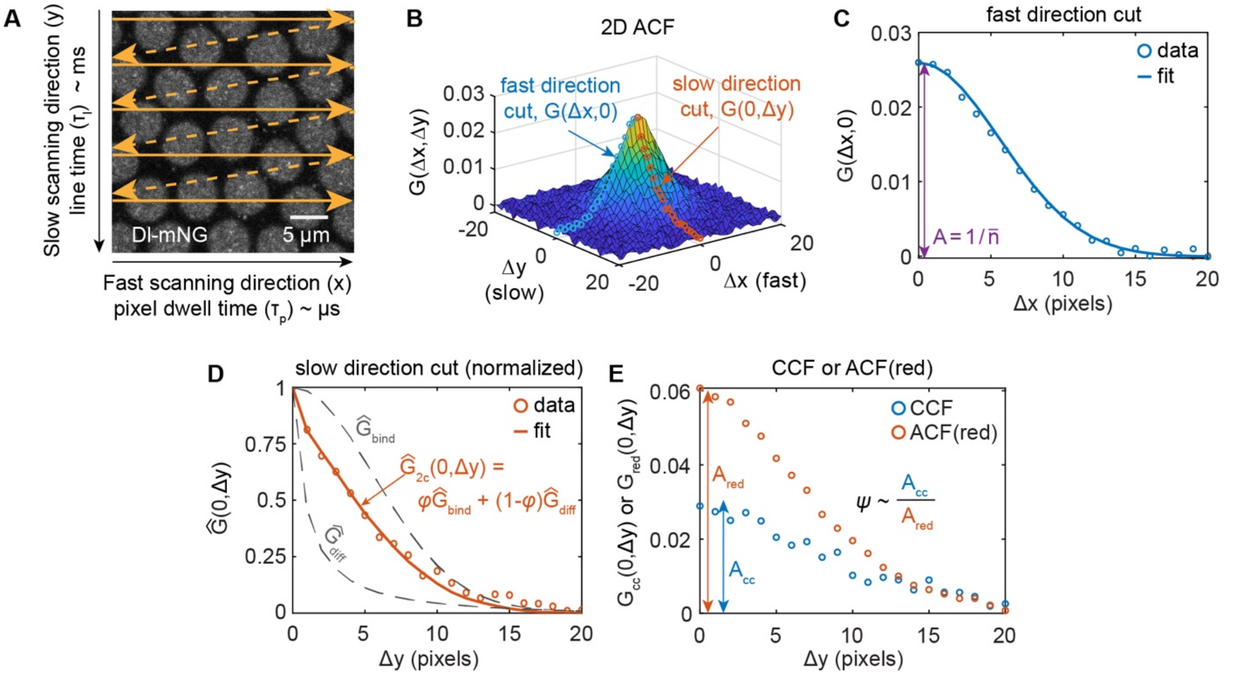

Figure S1. Raster Image Correlation Spectroscopy (RICS).

(A) Laser scanning confocal microscopes build images by raster scan, with a fast scanning direction (x direction, solid arrows), and a slow scanning direction (y direction) due to line retracing (dotted arrows). Image: mid-nc14 embryo expressing Dl-mNG.

(B) Two-dimensional ACF from embryo depicted in (A). Cuts along the fast (blue circles) and slow (orange circles) directions are depicted.

(C) Cut of ACF along the fast direction. Solid curve: fit of Gaussian-shaped PSF, used to estimate the ACF amplitude, A.

(D) Plot of the slow direction data (circles) and the fit to the slow direction (solid curve), composed of a linear combination between two ACFs (gray dotted curves): an immobile ACF ($\hat{G}_{bind}$) and a diffusible ACF ($\hat{G}_{diff}$). The linear combination weight is ϕ, the fraction immobile.

(E) Plots of cuts of the cross-correlation function (blue circles) and of the ACF in the red channel (orange circles). The ratio of the amplitudes (A_{cc}/A_{red}) is proportional to ψ, the fraction bound in active regions of the DNA.

The Figure and the caption were reproduced and modified from (*36*), distributed under the terms of the Creative Commons Attribution License, CC BY 4.0 (https://creativecommons.org/licenses/by/4.0).

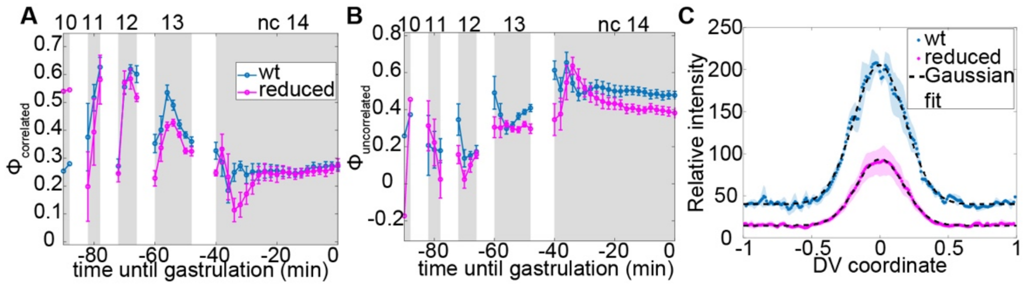

Figure S2: Dynamics of fraction of different pools of Dl from nc 10 until gastrulation for wt and reduced fly lines.

(A-B) Distribution of the populations including fraction of correlated population (A) and fraction of uncorrelated population (B).

Curves: mean values (n = 8 for wt embryos, n = 8 for reduced embryos). Error bars: S.E.M.

(C) Comparison of the Dl gradient in wt and reduced embryos (n = 3 for wt, n = 5 for reduced embryos). Shaded regions: S.E.M.

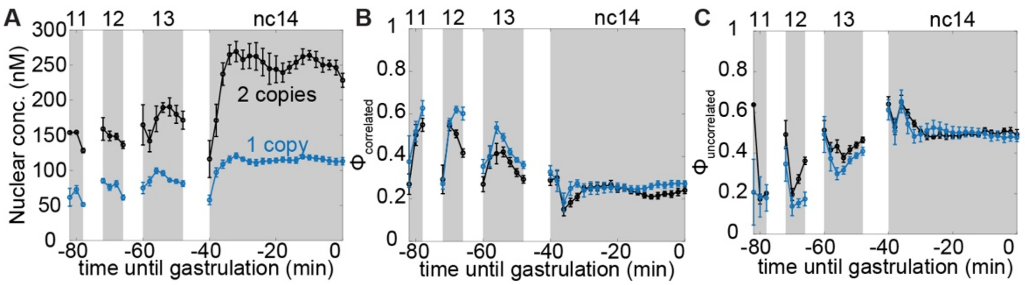

Figure S3: Comparison of the parameters in the ventral-most nuclei of embryos with one and two copies of Dl-mNG from nc 10 to gastrulation.

(A) Total nuclear concentration.

(B-C) Distribution of the populations including fraction of correlated population (B) and fraction of uncorrelated population (C).

Curves: mean values (n = 8 for embryos with 1 copy, n = 8 for embryos with 2 copies Dl-mNG). Error bars: S.E.M.

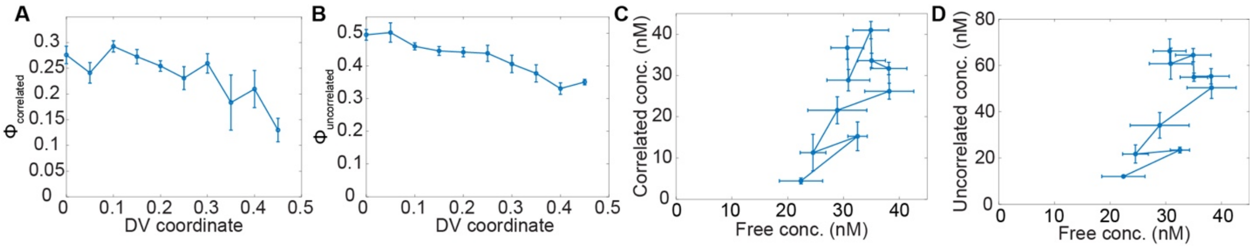

Figure S4: Variation of fraction of different pools of Dl mid nc 14 at different DV locations.

(A) Fraction of correlated population.

(B) Fraction of uncorrelated population.

(C-D) Dose/response map between free and the low-diffusivity populations including correlated population (C) and uncorrelated population (D).

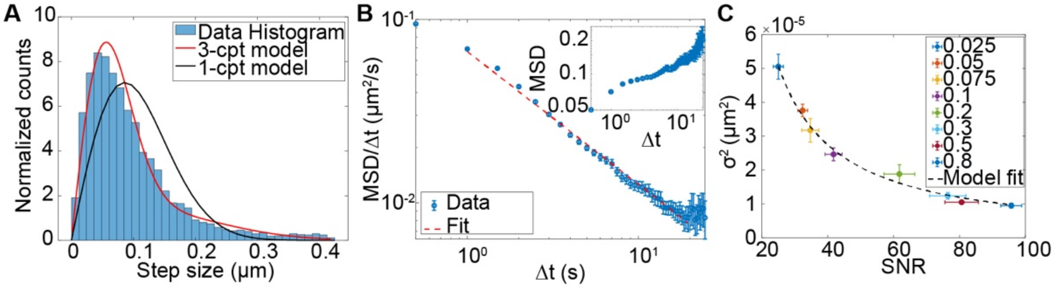

Figure S5: Model fits to the particle tracking parameters.

(A) one- and three-component model fitting for the determination of diffusivities and compositions of different populations.

(B) MSD analysis for the qualitative estimation of the diffusion regime of the population detected in the longer frame rate of 500 ms.

(C) Determination of localization precision using immobilized fluorescent beads imaged with varying laser powers. Each laser power was used to obtain 10 different acquisitions.

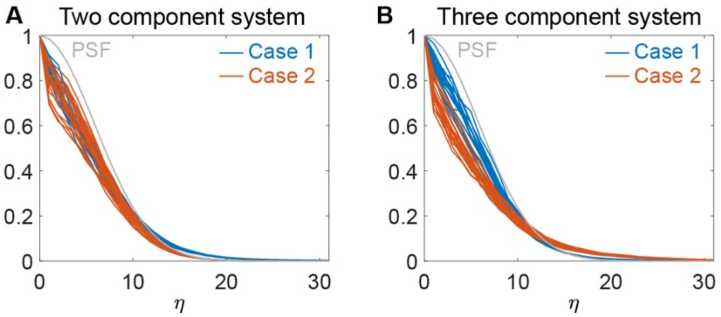

Figure S6: Simulations of individual ACFs.

(A) Twenty simulations of a two-component system for Case 1 (short line time; blue curves) and Case 2 (long line time; red curves).

(B) Twenty simulations of a three-component system for Case 1 (short line time; blue curves) and Case 2 (long line time; red curves).

Table S1: Phase separation propensity scores of Dl and mNG, the fluorescent protein tagged to it, using multiple algorithms.

Algorithm	Score for Dl	Score for mNG	Description
PSPredictor (*52*)	0.99	0.0815	Phase separation protein prediction score, highest being 1 and the lowest 0
PhaSePred (*53*)	0.93	-	Rank score, where highest rank score is 1 and the lowest is 0
FuzDrop (*54*)	0.99	0.2291	Probability of spontaneous liquid-liquid phase separation
PSPHunter (*55*)	0.81	0.55974	Probability to undergo phase separation

Movie Files

Movie S1: Image acquisition on the ventral side of the embryo from nc 10 until late nc 14 for RICS analysis. Mitosis not shown, as only interphase was used for the analysis. Multiple time series acquired by moving the ROI to different parts of the same embryo having identical DV coordinate to minimize photobleaching were concatenated. The repositioning is indicated by the appearance of a yellow dot on the top-right corner in the first few frames of the corresponding acquisition at the same location.

Movie S2: Detection of clusters that stay for multiple frames and the trajectories followed by them.

Movie S3: Image acquisitions showing Case 1 (short line time) and Case 2 (long line time) for the simultaneous detection of all three populations using RICS.
